# Colonization of mudflat substrate by microarthropods: the role of distance, inundation frequency and body size

**DOI:** 10.1007/s00442-024-05615-x

**Published:** 2024-09-04

**Authors:** Md Ekramul Haque, Maria Rinke, Ting-Wen Chen, Mark Maraun, Stefan Scheu

**Affiliations:** 1 J.F. Blumenbach Institute of Zoology and Anthropology, Department of Animal Ecology, University of Göttingen, Untere Karspüle 2, 37073 Göttingen, Germany; 2 Centre of Biodiversity and Sustainable Land Use, University of Göttingen, Büsgenweg 1, 37077 Göttingen, Germany

**Keywords:** Intertidal salt-marsh, Artificial islands, Dispersal, Environmental filtering, Community assembly

## Abstract

Salt marshes represent a unique ecosystem at the marine-terrestrial boundary of shallow protected coastlines. Microarthropods form an essential component of soil food webs, but how they colonize new intertidal habitats is little understood. By establishing two experimental systems without animals, we investigated microarthropod colonization (1) at the seashore from the pioneer zone to the lower and upper salt marsh and (2) at the same tidal height on artificial islands 500 m from the seashore. Potential source populations of microarthropods in the respective zones were also investigated. Colonization of microarthropods after 5 years was consistently faster on the seashore than on the artificial islands. Collembola and Mesostigmata colonized all the zones both on the seashore and on the artificial islands, with colonization being faster in the upper salt marsh and in the pioneer zone than in the lower salt marsh. Oribatida colonized the new habitats on the seashore, but only little on the artificial islands. Variations in species composition were more pronounced between salt marsh zones than between experimental systems, indicating that local environmental conditions (i.e., inundation frequency) are more important for the assembly of microarthropod communities than the distance from source populations (i.e., dispersal processes). Variations in community body size of Oribatida and Mesostigmata indicated environmental filtering of traits, with smaller species suffering from frequent inundations. Notably, Mesostigmata most successfully colonized the new habitats across salt marsh zones on both systems. Overall, the results document major mechanisms of colonization of intertidal habitats by microarthropods with different life histories and feeding strategies.

**Supplementary Information:**

The online version contains supplementary material available at 10.1007/s00442-024-05615-x.

## Introduction

Colonization involves the dispersal and establishment of organisms in new habitats. This fundamental process shapes the composition of ecological communities and biodiversity across landscapes. Colonization success is a product of two community assembly processes, dispersal and environmental filtering, with the former being a prerequisite for the latter. Colonization has been extensively studied in different habitats, including freshwater (Anderson and Smith 2004; Tavares et al. 2008; Incagnone et al. 2015), deep-sea (Grassle and Morse-Porteous 1987; Kelly et al. 2007; Priede and Froese 2013) and terrestrial ecosystems (Marsh et al. 2004; Bröring and Wiegleb 2005; Rosenberger et al. 2017). A wide range of taxa have been studied, including microbes (Wynn-Williams 1990; Vieira et al. 2020; Malard and Pearce 2022), plants (Wang et al. 2011; Lõhmus et al. 2014; García-Cervigón et al. 2017), vertebrates (Boyd and Pletscher 1999; Kerth and Petit 2005; Banaszek et al. 2010) and insects (Harrison 1989; Neve et al. 1996; Solbreck et al. 2023). However, information on colonization and community assembly processes of one of the most dynamic habitats, the interface between marine and terrestrial ecosystems, i.e. salt marshes, has received little attention.

Salt marshes occur at the marine-terrestrial interface along protected shallow coastlines throughout the world’s oceans. These unique habitats are characterized by halophytic plants (Allen 2000) and are influenced by regular tidal inundations (i.e., submergence due to water coverage during high tide). Formed by sediment accretion, salt marshes provide a gradient of bank elevation with associated differences in inundation frequency (Roozen and Westhoff 1985; Bockelmann et al. 2002; Caçador et al. 2007). Abiotic environmental conditions shape the system into distinct vegetation zones, such as the pioneer zone (PZ), the lower salt marsh (LSM) and the upper salt marsh (USM) (Petersen et al. 2014). Regular exposure to water level changes and episodic storms, especially in winter, make salt marshes a heterogeneous dynamic system. The sediment matrix, with its pore spaces, aggregates and organic matter, serves as an initial colonization site for terrestrial organisms. Plant and animal communities assemble along this dynamic gradient (Haynert et al. 2017; Winter et al. 2018; Lõhmus 2020).

In salt marsh systems, microarthropods with body sizes of a few millimetres (Karg 1993; Hopkin 1997; Weigmann 2006) are highly diverse and abundant (Polderman 1974; Sterzynska and Ehrnsberger 2000; Haynert et al. 2017). They play a critical role in ecosystem functions such as decomposition of organic matter, release of nutrients, formation of soil structure and stabilization of organic carbon (Rusek 1998; Grandy et al. 2016; Trentini et al. 2018). The dominant taxa of microarthropod communities in salt marshes are springtails (Collembola) and mites (Acari: Oribatida and Mesostigmata) (Polderman 1974; Sterzynska and Ehrnsberger 2000; Haynert et al. 2017). Collembola and Oribatida are decomposers that regulate microbial biomass and community composition, thereby indirectly influencing ecosystem functions (Griffiths and Bardgett 1997; Erktan et al. 2020a, b; Erktan et al. 2021). In food webs, Collembola contribute to the propagation of basal resources to higher trophic levels (Terborgh and Estes 2010; Sofo 2020). By contrast, Mesostigmata are dominant predators in salt marsh habitats and are likely to stabilize food webs against disturbance (Haynert et al. 2017). Collembola generally comprise r-selected taxa that disperse and reproduce rapidly (Petersen 2002), whereas Oribatida predominantly comprise K-selected taxa that develop and reproduce slowly (Crossley 1977; Maraun et al. 2003; Minor and Cianciolo 2007). The colonization of terrestrial habitats by Collembola, Oribatida and Mesostigmata typically follows successional changes and contributes to the formation of terrestrial ecosystems, which is particularly evident along the marine-terrestrial boundary (Scheu and Schulz 1996; Perez et al. 2013; Haynert et al. 2017). The colonization of salt marsh sediments by these microarthropod taxa is likely to play a crucial role in forming salt marsh food webs and stabilizing animal communities against disturbances.

Colonization of terrestrial habitats by microarthropods is a complex and dynamic process influenced by many factors, including individual dispersal ability, distance from source habitats and local environmental conditions. In salt marshes, microarthropod density and diversity have been shown to be reduced by tidal inundation (Haynert et al. 2017). Environmental conditions such as salinity, oxygen and food availability can influence colonization processes. However, different taxa may have specific tolerance ranges or traits for these environmental factors, resulting in different colonization patterns. Furthermore, as most microarthropods rely on passive water or wind dispersal (Hawes et al. 2007; Lehmitz et al. 2011; Schuppenhauer et al. 2019), smaller taxa may disperse longer distances than larger ones due to the lower mass and slower landing speed of the former (Jung and Croft 2001). Body size is therefore likely to be an important trait for microarthropod colonization of salt marsh habitats. However, few studies have investigated microarthropod colonization processes, with most studies conducted in the laboratory (Coleman and MacFadyen 1966; Hågvar and Abrahamsen 1980; Chauvat and Ponge 2002) and only few in the field (Dunger et al. 2002; Wanner and Dunger 2002; Stefano et al. 2007). Results suggest that microarthropods typically appear shortly after new land is exposed and act as foundation taxa in these habitats, facilitating vegetation succession and colonization by other organisms (Hodkinson et al. 2004). However, information on microarthropod colonization processes in the highly dynamic marine-terrestrial interface of salt marshes is limited. Our study fills this gap by investigating species composition, colonization success and body size of three dominant microarthropod taxa with different life histories and feeding strategies, i.e. Collembola, Oribatida and Mesostigmata, in salt marsh habitats of the Wadden Sea of Spiekeroog, Germany.

To understand colonization processes in salt marsh habitats with different exposure to tidal inundation and distance from the source habitats, two experimental systems were established: (1) new mudflat sediments devoid of microarthropods exposed across salt marsh zones of different inundation frequency, i.e. the USM, LSM and PZ, on Spiekeroog Island, and (2) artificial islands erected in the tidal flats about 500 m from the seashore, where the same experimental design was established (i.e., new mudflat sediment exposed at the level of the USM, LSM and PZ). We compared the colonization patterns of the three microarthropod taxa in the different salt marsh zones between the two experimental systems, allowing us to differentiate between short- and long-distance dispersal (i.e., effects of dispersal limitation), while the different inundation frequencies of the USM, LSM and PZ allowed us to compare the strength of environmental filtering by inundation between these taxa. We hypothesized that (1) microarthropod colonization of newly exposed mudflat sediments is faster on Spiekeroog Island than on artificial islands; (2) assuming that long-distance dispersal is mainly due to wind rather than water, microarthropods of the less inundated USM disperse most easily; (3) differences in microarthropod community composition are more pronounced between salt marsh zones than between experimental systems. In addition, we hypothesized that (4) microarthropod colonization success is mediated by body size, but the pattern varies between taxa.

## Materials and methods

### Study site

The study was conducted in the salt marshes of the Wadden Sea of Spiekeroog, Germany. The Wadden Sea is the largest uninterrupted tidal flat in the world, extending along the coasts of the Netherlands, Germany and Denmark (Reise et al. 2010; Kabat et al. 2012), and is home to back-barrier island salt marshes (Adam 2002; Bakker et al. 2015; Winter et al. 2018). The island of Spiekeroog is one of the East Frisian barrier islands off the North Sea coast and is part of the Wadden Sea National Park (53°45′02"—53°47′01" N and 07°40′00"—07°49′01" E). Salt marshes and mudflats are located on the southern leeward side of Spiekeroog Island. As the leeward side of the island is sheltered from strong winds and tidal waves (Bakker et al. 2015), sediment accumulates gradually and over time a gradient of shore height from land to sea is formed (Bakker et al. 2015; Haynert et al. 2017). Shore height is related to inundation frequency and associated abiotic conditions (i.e., soil salinity, anoxia and waterlogging). This results in distinct salt marsh zones characterized by unique vegetation, i.e. the USM, LSM and PZ (Bockelmann et al. 2002; Silvestri et al. 2005; Haynert et al. 2017). The USM is situated 35 cm above the mean high water level, inundated 35–70 times per year and dominated by *Elymus athericus* with a soil salinity of 5–20%; the LSM is situated 0–35 cm above the mean high water level, inundated 150–250 times a year and dominated by *Atriplex portulacoides* and *Puccinellia maritima* with a soil salinity of 20–26%; the PZ lies below the mean high water level, is inundated twice a day and dominated by *Salicornia stricta* and *Spartina anglica* with a soil salinity of 26–32% (Bockelmann et al. 2002; Niedrighaus 2009; Haynert et al. 2017). The distinct vegetation and salt marsh zones are associated by pronounced habitat heterogeneity on small spatial scales. Therefore, salt marsh habitats provide an ideal model system to study colonization and assembly processes of animal communities.

### Design of the colonization experiment

**Fig. 1:**
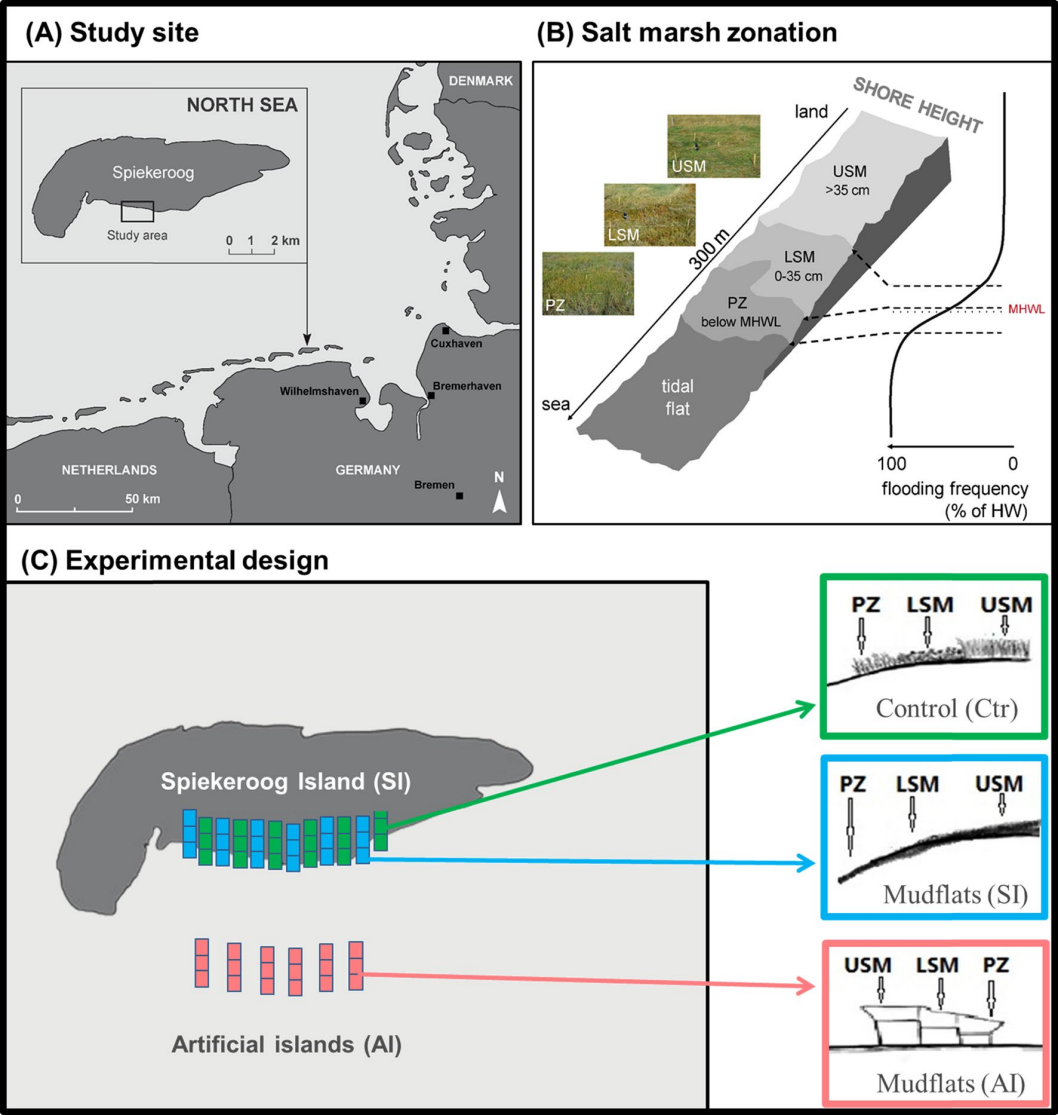
Location of the study site in the Wadden Sea of Germany (**A**), schematic representation of the salt marsh zonation (**B**) and the design of the colonization experiment (**C**). *USM* upper salt marsh, *LSM* lower salt marsh, *PZ* pioneer zone. The control systems (Ctr) consisted of the natural salt marsh vegetation on the Spiekeroog Island. The map of the study site and the salt marsh zonation was adopted from Haynert et al. (2017)

The experimental study system was established within the framework of the DynaCom project (https://uol.de/en/icbm/collaborative-projects/dynacom), which aims at investigating intertidal metacommunities (Balke et al. 2017), in particular the establishment and dispersal-mediated assembly of communities (Lõhmus et al. 2020; Yong et al. 2022). In 2014, two experimental salt marsh systems consisting of mudflat sediments without terrestrial animals were established. The first experimental system was established along the marine-terrestrial boundary from the PZ to the LSM to the USM on Spiekeroog Island (SI system). The second was established on artificial islands (AI system) about 500 m from the seashore. The artificial islands consisted of metal cages filled with salt marsh sediment and exposed at three heights corresponding to the PZ, LSM and USM. Both experimental systems were filled with sediment from the tidal flats bare of any terrestrial animals and left for spontaneous colonization. Prior to the addition of the tidal flat sediment, the plots of the SI system were cleared of soil and vegetation to a depth of 30 cm and the sediment was then added to the level of the surrounding soil. In addition, control plots with natural salt marsh vegetation were established at the PZ, LSM and USM on Spiekeroog Island (Ctr system). In each of the three salt marsh systems, six replicate plots of 90 cm × 90 cm were established in each of the three salt marsh zones, resulting in a total of 54 plots (Fig. 1**)**.

### Sampling and identification of microarthropods

In 2019, 5 years after the establishment of the experiment, microarthropods were sampled from the three salt marsh systems. Four soil cores (⌀ 5 cm, 5 cm depth) were collected from each of the 54 plots, resulting in 216 samples. The soil cores were stored in plastic containers and transported to the laboratory at the University of Göttingen. Microarthropods were extracted in a mixture of ethylene glycol and water (1:1) using a high gradient canister technique (Kempson et al. 1963). Microarthropods were filtered through 45 µm mesh, washed with water and preserved in 70% ethanol prior to sorting, counting and species identification under the microscope. For species identification we used Hopkin (1997) for Collembola, Weigmann (2006) for Oribatida and Karg (1989, 1993) for Mesostigmata.

### Statistical analysis

All statistical analyses were performed in R 4.3.1 (R Core Team 2023). Data from the four individual soil cores per plot were merged to obtain plot-level data and used as the community unit of analysis. Abundance data were expressed in individuals per square meter (i.e., density). Analysis of variance (ANOVA) was used to compare species richness and abundance of microarthropod communities between salt marsh zones and between experimental systems. Prior to ANOVA, abundance data were log-transformed to improve the normal distribution and to meet the assumptions of parametric testing. Community composition of the three microarthropod taxa across salt marsh zones and experimental systems was analysed by non-metric multidimensional scaling (NMDS) based on the Bray–Curtis distance matrix (Bray and Curtis 1957) using the “*metaMDS*” function in the “*vegan*” package (Oksanen et al. 2022). Differences in community composition of the three microarthropod taxa between salt marsh zones and experimental systems were analysed by permutational multivariate analysis of variance (PERMANOVA) using the same distance method (i.e., Bray–Curtis) in the “*adonis2*” function (McArdle and Anderson 2001) of the “*vegan*” package (Oksanen et al. 2022). In addition, the Mahalanobis distance between communities was calculated to examine differences in species composition.

Colonization success between zones of the experimental salt marsh systems were compared between microarthropod taxa. Soil cores were scored as either 1 or 0 for the presence or absence of individual taxa. The sum of the scores of the four soil cores taken per plot was used to represent the presence-absence score of microarthropods in that plot. The colonization success of each plot of the SI and AI systems was calculated as the ratio of the presence-absence scores of microarthropods in the experimental salt marsh systems to the corresponding scores in the control (see Supplementary Material Table S4 for the colonization success of each plot**)**. Using the interquartile range criterion, one outlier of the Mesostigmata community was detected and removed from the analysis. ANOVA was performed to test whether colonization success differed between microarthropod taxa across the zones of the experimental salt marsh systems, followed by a post-hoc test (Tukey’s HSD). Normality of residuals and equality of variances were checked using the Shapiro–Wilk test (Shapiro and Wilk 1965) and the Levene test (Levene 1960), respectively.

To test whether the colonization success of microarthropods correlated with their body size, correlation coefficients were calculated between community colonization success and community-weighted mean (CWM) body size using the “*Kendall*” method (Kendall 1938) in the “*cor.test”* function. Mean body sizes of Oribatida and Mesostigmata species were obtained from the literature (Karg 1989; 1993; Weigmann 2006); Collembola body sizes were obtained from the #GlobalCollembola trait database (T.-W. Chen, unpubl. data). For the mean body size of the species see Supplementary Material Table S1. CWM body size was calculated using the “*cwm*” function in the “*BAT*” package (Cardoso et al. 2022). To assess the contribution of size-related filtering processes to community assembly of the three microarthropod taxa, variation in CWM body size across zones and salt marsh systems was analysed using ANOVA.

## Results

### Species richness and abundance

A total of 4499 adult individuals belonging to 29 families, 44 genera and 51 species were identified, including 12 Collembola, 14 Oribatida and 25 Mesostigmata species (Supplementary Material Table S2). The colonization of the two experimental salt marsh systems by microarthropods was more pronounced in the SI system than in the AI system, regardless of taxa. In the SI system, a total of 10 (83.3%) Collembola species, 12 (85.7%) Oribatida species, including one endemic species (i.e., not found in the other salt marsh zones and experimental systems), and 22 (88.0%) Mesostigmata species, including two endemic species, were able to colonize. In the AI system, 6 (50.0%) Collembola species, 2 (14.3%) Oribatida species, and 9 (36.0%) Mesostigmata species, including one endemic species, were able to colonize (Supplementary Material Table S2, Figure S1).

**Fig. 2:**
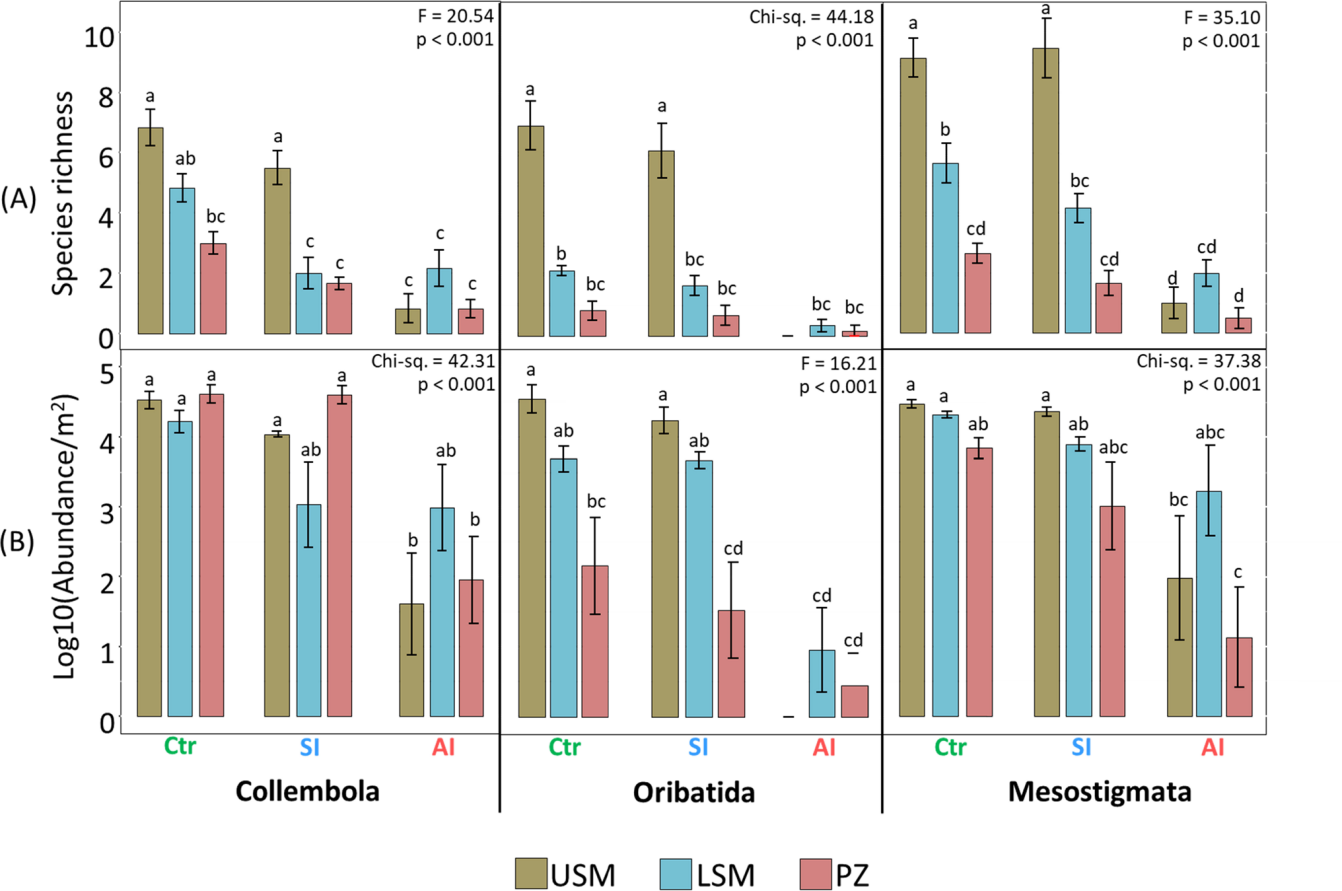
Mean ± standard error of species richness (**A**) and abundance per square meter (**B**) of Collembola, Oribatida and Mesostigmata in the upper salt marsh (USM), lower salt marsh (LSM) and pioneer zone (PZ) in the three experimental systems, i.e. control sites on the Spiekeroog Island (Ctr), experimental salt marsh systems on the Spiekeroog Island (SI) and on artificial islands (AI). Treatments with the same letter are not significantly different at p < 0.05

Overall, species richness was highest in the Ctr system and lowest in the AI system, regardless of microarthropod taxa (Fig. 2A). Species richness of all three taxa decreased consistently from the USM to LSM to PZ in the Ctr and SI systems, whereas in the AI system Collembola and Mesostigmata species richness in the LSM exceeded that in the USM and PZ. No Oribatida were found in the USM of the AI system (Fig. 2A). Similar to species richness, total microarthropod abundance was consistently highest in the Ctr system and lowest in the AI system, regardless of taxa. Microarthropod abundance decreased from the USM to the LSM to the PZ in the Ctr and SI systems, with the exception of Collembola being of similar abundance across salt marsh zones (Fig. 2B). However, in the AI system, the abundance of Collembola and Mesostigmata in the LSM exceeded that in the USM and PZ.

### Community composition

Community composition of Collembola and Mesostigmata varied significantly between salt marsh zones and experimental systems (Table 1). For Oribatida, community composition also varied significantly between salt marsh zones, whereas differences between experimental systems were not significant (presumably due to their generally low colonization of the AI system; see below). In general, variation in community composition of each of the three microarthropod taxa was more pronounced between salt marsh zones than between experimental systems.

**Table 1:** Community composition of microarthropod taxa (Collembola, Oribatida, Mesostigmata) explained by experimental systems (control system on the Spiekeroog Island, and experimental systems on the Spiekeroog Island and artificial islands) and salt marsh zones (upper salt marsh, lower salt marsh, pioneer zone) according to PERMANOVA. Significant effects at p < 0.05 are shown in bold

Microarthropods	Experimental system (E)			Salt marsh zone (Z)			E x Z		
	R^2^	Pseudo-F	p-value	R^2^	Pseudo-F	p-value	R^2^	Pseudo-F	p-value
Collembola	0.14	3.5	** < 0.001**	0.19	5.3	** < 0.001**	0.45	3.9	**< 0.001**
Oribatida	0.07	1.2	0.27	0.33	7.8	** < 0.001**	0.57	4.9	**< 0.001**
Mesostigmata	0.12	2.7	**0.001**	0.20	5.2	** < 0.001**	0.44	3.5	** < 0.001**

**Fig. 3:**
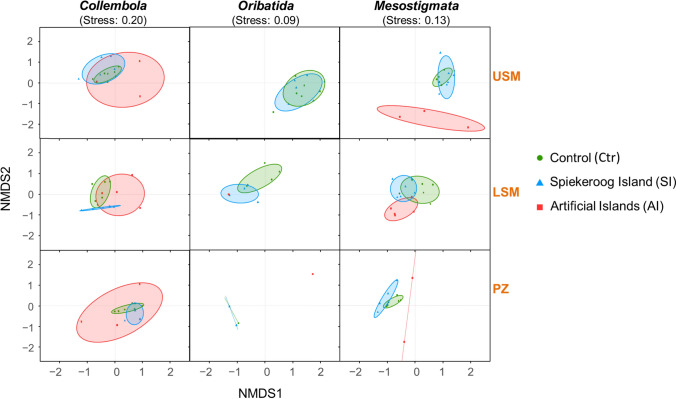
Variations in species composition of microarthropod taxa (Collembola, Oribatida, and Mesostigmata) between experimental systems (control system on the Spiekeroog Island and experimental systems on the Spiekeroog Island and artificial islands) and salt marsh zones (upper salt marsh, lower salt marsh and pioneer zone). Ellipses represent 75% confidence intervals

Microarthropod communities across salt marsh zones in the SI system were more similar to those in the Ctr system than those in the AI system, regardless of microarthropod taxa (Fig. 3; for pairwise differences in community composition see Supplementary Material Table S3**)**. Collembola communities of each of the three systems overlapped, but the confidence intervals of the AI system were consistently larger than those of the SI and Ctr systems across salt marsh zones. For Oribatida, only few individuals occurred in the LSM and PZ of the AI system, whereas community composition in the SI system was similar to that of the Ctr system, especially in the USM. By contrast, Mesostigmata communities in the AI system formed separate clusters from those of the SI and Ctr systems in each of the salt marsh zones.

### Colonization success and relationship with body size

**Fig. 4:**
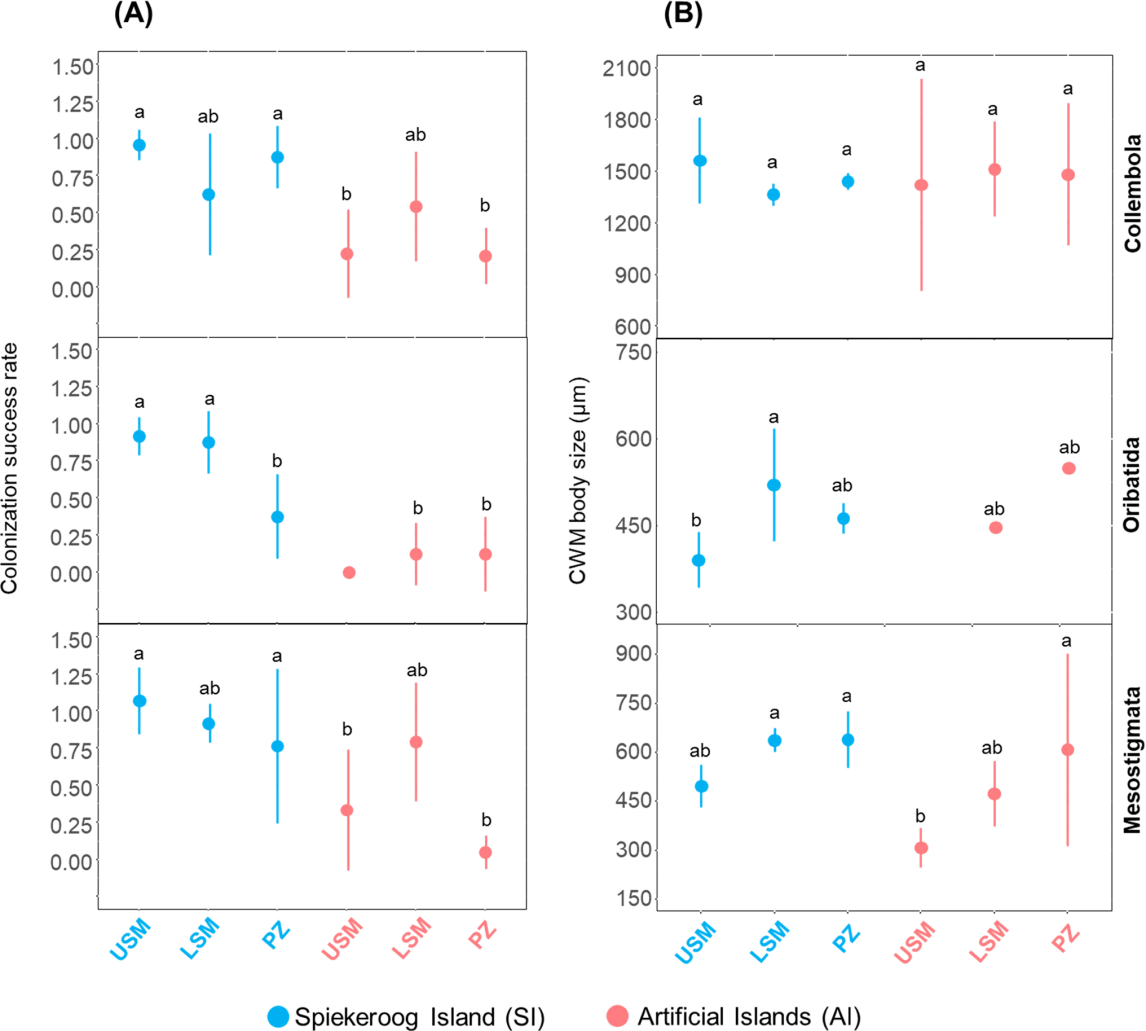
Mean ± standard deviation of colonization success rate (A) and community-weighted mean (CWM) body size (B) of Collembola, Oribatida and Mesostigmata in the upper salt marsh (USM), lower salt marsh (LSM) and pioneer zone (PZ) of the Spiekeroog Island (SI) and artificial island (AI) systems. Treatments with the same letter are not significantly different at p < 0.05

Similar to microarthropod community composition, colonization was generally more complete in the SI than in the AI system (Fig. 4A**, **Table 2). Between the three microarthropod taxa, colonization was most successful in Mesostigmata, followed by Collembola and Oribatida. For Oribatida, colonization success in the SI system was less complete in the PZ than in the USM and LSM.

**Table 2:** Differences in colonization success rate and community-weighted mean (CWM) body size of microarthropod taxa (Collembola, Oribatida, Mesostigmata) between experimental salt marsh systems (the Spiekeroog Island and artificial islands) and between salt marsh zones (upper salt marsh, lower salt marsh, pioneer zone) as indicated by ANOVA. Significant effects at p < 0.05 are shown in bold

Parameters	Microarthropods	Experimental system (E)		Salt marsh zone (Z)		E x Z	
		F	p-value	F	p-value	F	p-value
Colonization success rate	Collembola	27.5	**< 0.001**	0.1	0.9	4.8	**0.01**
	Oribatida	103.3	**< 0.001**	4.4	**0.02**	7.5	**0.002**
	Mesostigmata	19.2	** < 0.001**	4.8	**0.01**	3.0	0.06
CWM body size	Collembola	0.02	0.8	0.2	0.8	0.5	0.6
	Oribatida	0.3	0.6	4.8	**0.02**	2.7	0.1
	Mesostigmata	13.04	**0.001**	9.1	**0.001**	1.3	0.3

CWM body size of Oribatida and Mesostigmata, but not of Collembola, varied significantly between salt marsh zones of the experimental systems (Table 2). For both Oribatida and Mesostigmata it increased from the USM to the LSM and partially to the PZ (Fig. 4B). Colonization success was not significantly correlated with CWM body size, regardless of microarthropod taxa (Supplementary Material Table S2).

## Discussions

We studied the colonization patterns of mudflat sediments by Collembola, Oribatida and Mesostigmata in experimental salt marsh systems consisting of natural seashore (Spiekeroog Island) and artificial habitats (man-made islands about 500 m from the seashore). We hypothesized that (1) microarthropod colonization of newly exposed mudflat sediments is faster in the SI system than in the AI system, (2) species of the less frequently inundated USM disperse most easily, (3) variation in microarthropod community composition is more pronounced between salt marsh zones than between experimental systems, and (4) colonization success varies between microarthropod taxa and is related to body size.

Mudflat sediment colonization by microarthropods was consistently faster and more complete in the SI than in the AI system, regardless of microarthropod taxa, supporting the first hypothesis. The experimental mudflat plots in the SI system were surrounded by intact salt marsh, which served as a source habitat, allowing microarthropods to colonize the mudflat material by both active movement and passive dispersal by tide and wind. By contrast, the artificial islands were placed in the Wadden Sea tidal flats about 500 m from the source habitats on Spiekeroog Island. Active dispersal of flightless microarthropods from the source habitats to the AI system is unlikely; rather, they are passively dispersed over large distances by tides, wind and birds. Floating in water and rafting on plant debris or wrack during tidal surges may facilitate long-distance dispersal of microarthropods, as demonstrated for Oribatida across distant oceanic islands or even continents (Schatz 1991, 1998; Schuppenhauer et al. 2019; Lindo 2020). Microarthropods are also dispersed by wind, allowing them to colonize new habitats (Lindroth 1973; Thornton et al. 1988; Schneider et al. 2011). In addition, phoresy on birds is a way for Oribatida to bridge long distances (Krivolutsky and Lebedeva 2002; Krivolutsky et al. 2004; Lebedeva 2012). Although birds heavily colonize salt marshes (Dierschke 2002; Dierschke and Bairlein 2004), it is unlikely that they transported Oribatida to the AI system as no Oribatida were found in the USM. The results suggest that long-distance dispersal is relatively slow, but could mediate local colonization of new intertidal habitats.

Of the three microarthropod taxa, Oribatida were the least effective colonizers in both the SI and AI systems. In particular in the AI system, colonization by passive dispersal of Oribatida was less pronounced than that of Collembola and Mesostigmata, suggesting that Oribatida require longer periods of time (more than 5 years in our study) to colonize new intertidal habitats. The less effective colonization of the SI system by Oribatida compared to the other two taxa further indicates slow active dispersal of Oribatida. They move in the range of about 5 cm per day (Ojala and Huhta 2001; Lehmitz et al. 2012; Cameron et al. 2013), whereas Collembola can actively bridge distances > 3 m per day (Hågvar 1995; Zettel et al. 2000; Åström and Bengtsson 2011; Ponge 2020). Similarly, Mesostigmata are surface-living taxa that actively hunt for prey and may bridge long distances by active movement. Phoresy via ground beetles is also likely to contribute to the rapid colonization of Mesostigmata in both the SI and AI systems (Gwiazdowicz and Gutowski 2012). Furthermore, the high mobility and surface-living of Collembola and Mesostigmata suggests that they are more susceptible to displacement by water and wind than Oribatida which more intensively colonize the soil. To drift on the water surface during passive dispersal, many Collembola species are osmoconformers and can survive at high salinity (Witteveen et al. 1987; Witteveen and Joosse 1988). Many Mesostigmata species can also survive prolonged submersion. By contrast, most intertidal Oribatida species have large tarsal claws, allowing them to withstand tidal forces by clinging to plants or other substrates (Pfingstl 2023) presumably contributing to low dispersal by water. Although Oribatida can be dispersed up to a few hundred meters by wind, most wind dispersed species are arboreal (Lehmitz et al. 2011). Very limited wind dispersal of Oribatida is supported by the fact that they did not colonize the USM in the AI system. The lower colonization of the PZ by Oribatida in the AI system, however, may also have been due to their lower density in the source habitats on Spiekeroog Island than that of the other two taxa.

Microarthropod colonization was faster in the USM and PZ than in the LSM for both SI and AI systems, supporting our second hypothesis. The USM is similar to terrestrial systems and is only inundated during storm events. Microarthropods in the USM can intensively and actively colonize adjacent habitats. Microarthropods in the USM are also more intensively exposed to wind and are likely to be passively dispersed by wind rather than water. By contrast, the PZ resembles aquatic habitats with frequent inundations, which limits active movement of microarthropods but increases passive dispersal by water. The general decrease in Oribatida density from the USM to the LSM to the PZ reflects the increased disturbance by tides, which fill the habitat by salt water. In our study, only few Oribatida species colonized the most frequently inundated PZ, such as *Ameronothrus schneideri*, *Zachvatkinibates quadrivertex* and *Liebstadia similis*. These species are halobionts able to tolerate salinity and survive submersion (Schulte 1973; Polderman 1974; Pfingstl 2013). More species of Collembola and Mesostigmata than Oribatida in the PZ indicate a greater number of species in the former groups that can cope with saline conditions and frequent inundations. Most of these Collembola (*Archisotoma besselsi*, *Halisotoma maritima*, *Thalassaphorura debilis*, *Mesaphorura krausbaueri*, *Mesaphorura macrochaeta* and *Parisotoma notabilis*) and Mesostigmata species (*Dendrolaelaps halophilus*, *Cheiroseius necorniger*, *Vulgarogamasus* cf*. trouessarti* and *Gaeolaelaps praesternalis*) are semi-aquatic, salt-tolerant and trophic specialists (Salmane 2000; Haynert et al. 2017).

In support of our third hypothesis, microarthropod community compositions generally varied more between salt marsh zones than between experimental systems, although variation was best explained by both factors. Mesostigmata species compositions varied significantly between salt marsh zones and between experimental systems. Collembola communities overlapped most between experimental systems across salt marsh zones, with more heterogeneous composition in the AI system than in the SI and control systems. Community composition of Oribatida varied significantly between salt marsh zones and, as indicated by only few records, colonization of the artificial islands by Oribatida was very limited. These results suggest that differences in local environmental conditions due to tidal inundation frequency (suggesting habitat filtering processes) between salt marsh zones are more important than geographical distance to source populations (suggesting dispersal limitation) for the assembly of salt marsh microarthropod communities, particularly for Oribatida.

As the frequency of tidal inundation increases from the USM towards the PZ, the pore space in the mudflat matrix in the twice-daily inundated PZ remains largely filled with water likely affecting the abundance and composition of microarthropod communities (Zaitsev and Wolters 2006; Nielsen et al. 2008; Erktan et al. 2020a, b). Limited pore space is likely to more strongly affect small-sized taxa colonizing soil pores than larger taxa predominantly colonizing the soil surface. Supporting this assumption, the CWM body size of Oribatida and Mesostigmata increased from the USM to the PZ, although the CWM body size was not significantly correlated with colonization success. This suggests that inundation frequency favours larger individuals after they have colonized new habitats. In contrast to Oribatida and Mesostigmata, Collembola CWM body size did not vary between salt marsh zones, suggesting that body size is not a key trait for Collembola in their post-colonization processes. At our study sites, most Collembola species are larger than mites and live predominantly on the surface rather in soil pores, and this likely contributed to their high density in the PZ. Collembola may use resources on the soil surface more efficiently than the other two taxa (Sterzynska and Ehrnsberger 2000; Mertens et al. 2007; Haynert et al. 2017). Further, they may recover faster from flood-related mortality due to rapid reproduction (Adis and Junk 2002; González‐Macé and Scheu 2018) and the production of flood-tolerant eggs (Tamm 1984; Marx et al. 2012). In addition, Collembola are physiologically adapted to waterlogged habitats and can adjust their metabolism under anoxia (Zinkler and Platthaeus 1996). They may also trap air bubbles as a potential behavioural trait (Zinkler et al. 1999). The observed difference in CWM body size between the two mite taxa and Collembola reflect differences in trait-based assembly processes after colonization in salt marshes, partially supporting our fourth hypothesis.

Colonization by microarthropods varied significantly between zones of the experimental salt marsh systems and was higher in the SI than in the AI system. This suggests that both distance from the source habitats and changes in the local environment related to inundation frequency structure microarthropod communities. In terms of community assembly processes, dispersal limitation (suggested by the distance of the AI system from the seashore) is most pronounced in Oribatida, less in Mesostigmata and least in Collembola. However, for all the three taxa, post-colonization filtering processes (mainly by inundation) are more important than dispersal per se.

## Conclusions

Our unique experimental systems allowed new insights into the patterns and processes of colonization of intertidal salt marshes by microarthropods, and comparisons between the importance of dispersal and body size-related filtering processes. Differences in the colonization patterns of microarthropod taxa between salt marsh zones indicate stronger dispersal limitation in Oribatida than in Collembola and Mesostigmata. Greater variation in species composition between salt marsh zones than between experimental systems indicates that local environmental conditions (i.e., inundation frequency) are more important for microarthropod community assembly than distance (i.e., dispersal) from source populations. This is further supported by the increase in CWM body size of Oribatida and Mesostigmata from the USM to the PZ. Overall, the colonization processes of intertidal habitats by microarthropods are mainly driven by environmental filtering in addition to dispersal, with the latter varying between taxa.

## Supplementary Information

Below is the link to the electronic supplementary material.

Supplementary file1 (DOCX 1339 KB)

## Data Availability

The datasets used and analysed in the study are available on request from the corresponding author.

## Code availability

The R codes used in the analysis are available from the corresponding author upon request.
