# Supplementary material for: Colonization of mudflat substrate by microarthropods: the role of distance, inundation frequency and body size
Source: Oecologia. 2024 Sep 4;206(1-2):87–100. doi: 10.1007/s00442-024-05615-x (PMC11489214; doi:10.1007/s00442-024-05615-x)
Supplement: Supplementary file 1 — Supplementary file1 (DOCX 1339 KB) [file 442_2024_5615_MOESM1_ESM.docx]

**Supplemental materials for the manuscript:**

**Title: Colonization of mudflat substrate by microarthropods: the role of distance, inundation frequency and body size**

**Authors:** Md Ekramul Haque, Maria Rinke, Ting-Wen Chen, Mark Maraun, & Stefan Scheu

**Corresponding author:** Ting-Wen Chen ([tchen2@gwdg.de](mailto:tchen2@gwdg.de))

**Table S1.** List of microarthropod species with mean body size. Body size data were obtained from the #GlobalCollembola trait database (T.-W. Chen, unpubl. data), Weigmann (2006) and Karg (1989, 1993) for Collembola, Oribatida and Mesostigmata, respectively.

| **Microarthropod taxa** | **No** | **Family name** | **Name of species** | **Average**  **body size (***μm***)** |
| --- | --- | --- | --- | --- |
| Collembola | 1 | Isotomidae | *Archisotoma besselsi* | 1427 |
|  | 2 | Isotomidae | *Halisotoma maritima* | 2036 |
|  | 3 | Isotomidae | *Parisotoma notabilis* | 1040 |
|  | 4 | Isotomidae | *Isotoma viridis* | 4200 |
|  | 5 | Onychiuridae | *Thalassaphorura debilis* | 1430 |
|  | 6 | Tullbergiidae | *Mesaphorura macrochaeta* | 648 |
|  | 7 | Tullbergiidae | *Mesaphorura krausbaueri* | 647 |
|  | 8 | Entomobryidae | *Entomobrya lanuginosa* | 1873 |
|  | 9 | Neanuridae | *Friesea mirabilis* | 1578 |
|  | 10 | Hypogastruridae | *Schoettella ununguiculata* | 1405 |
|  | 11 | Hypogastruridae | *Willemia anophthalma* | 642 |
|  | 12 | Hypogastruridae | *Hypogastrura sahlbergi* | 1944 |
| Oribatida | 13 | Oppiidae | *Multioppia neglecta* | 325 |
|  | 14 | Oppiidae | *Ramusella clavipectinata* | 337 |
|  | 15 | Oppiidae | *Dissorhina ornate* | 300 |
|  | 16 | Oppiidae | *Microppia minus* | 192 |
|  | 17 | Tectocepheidae | *Tectocepheus velatus subsp. sarekensis* | 327 |
|  | 18 | Liebstadiidae | *Liebstadia similis* | 550 |
|  | 19 | Punctoribatidae | *Zachvatkinibates quadrivertex* | 447 |
|  | 20 | Hermanniidae | *Hermannia pulchella* | 975 |
|  | 21 | Scheloribatidae | *Scheloribates laevigatus* | 595 |
|  | 22 | Thyrisomidae | *Banksinoma lanceolata* | 330 |
|  | 23 | Ceratozetidae | *Trichoribates incisellus* | 487 |
|  | 24 | Ameronothridae | *Ameronothrus schneideri* | 562 |
|  | 25 | Oribatulidae | *Oribatula tibialis* | 470 |
|  | 26 | Phenopelopidae | *Eupelops occultus* | 520 |
| Mesostigmata | 27 | Uropodidae | *Uropoda repleta* | 690 |
|  | 28 | Digamasellidae | *Dendrolaelaps halophilus* | 610 |
|  | 29 | Digamasellidae | *Dendrolaelaps arenarius* | 250 |
|  | 30 | Blattisociidae | *Cheiroseius necorniger* | 647 |
|  | 31 | Ascidae | *Leioseius salinus* | 330 |
|  | 32 | Ascidae | *Arctoseius cetratus* | 275 |
|  | 33 | Ascidae | *Gamasellodes bicolor* | 270 |
|  | 34 | Rhodacaridae | *Rhodacarus reconditus* | 380 |
|  | 35 | Rhodacaridae | *Rhodacarus salarius* | 520 |
|  | 36 | Rhodacaridae | *Rhodacarellus silesiacus* | 280 |
|  | 37 | Pachylaelapidae | *Pachylaelaps cf. siculus* | 535 |
|  | 38 | Halolaelapidae | *Halolaelaps (Saprogamasellus) strenzkei* | 400 |
|  | 39 | Laelapidae | *Pseudoparasitus dentatus* | 621 |
|  | 40 | Laelapidae | *Pseudoparasitus cf. centralis* | 550 |
|  | 41 | Laelapidae | *Gaeolaelaps praesternalis* | 397 |
|  | 42 | Laelapidae | *Gaeolaelaps aculeifer* | 602 |
|  | 43 | Laelapidae | *Cosmolaelaps vacuus* | 445 |
|  | 44 | Veigaiidae | *Cyrthydrolaelaps incisus* | 535 |
|  | 45 | Parasitidae | *Paragamasus runciger* | 782 |
|  | 46 | Parasitidae | *Vulgarogamasus cf. trouessarti* | 1200 |
|  | 47 | Parasitidae | *Pergamasus crassipes* | 810 |
|  | 48 | Parasitidae | *Pergamasus celticus* | 673 |
|  | 49 | Phytoseiidae | *Amblyseius neobernhardi* | 357 |
|  | 50 | Phytoseiidae | *Proprioseiopsis sp. cf. okanagensis* | 425 |
|  | 51 | Macrochelidae | *Macrocheles glaber* | 670 |

**Table S2.** Colonization of upper salt marsh (USM), lower salt marsh (LSM) and pioneer zone (PZ) of the natural salt marsh control system (Ctr) and the experimental Spiekeroog Island (SI) and artificial island (AI) systems by Collembola, Oribatida, Mesostigmata.

|  | **Total no. of species** | **No. of species in different salt marsh zones and experimental systems** | | | | | | | | | | | |
| --- | --- | --- | --- | --- | --- | --- | --- | --- | --- | --- | --- | --- | --- |
|  |  | **Ctr** | | | | **SI** | | | | **AI** | | | |
|  |  | Total | USM | LSM | PZ | Total | USM | LSM | PZ | Total | USM | LSM | PZ |
| **Collembola** | **12** | **12** | **12** | **8** | **6** | **10** | **10** | **4** | **3** | **6** | **4** | **6** | **4** |
| % of the total species |  | 100 | 100 | 66.7 | 50 | 83.3 | 83.3 | 33.3 | 25 | 50 | 33.3 | 50 | 33.3 |
| No. of endemic species* |  | 2 | 1 | 0 | 0 | 0 | 0 | 0 | 0 | 0 | 0 | 0 | 0 |
| **Oribatida** | **14** | **13** | **11** | **4** | **2** | **12** | **11** | **3** | **2** | **2** | **-** | **1** | **1** |
| % of the total species |  | 92.9 | 78.6 | 28.6 | 14.3 | 85.7 | 78.6 | 21.4 | 14.3 | 14.3 | - | 7.1 | 7.1 |
| No. of endemic species* |  | 2 | 1 | 1 | 0 | 1 | 1 | 0 | 0 | 0 | - | 0 | 0 |
| **Mesostigmata** | **25** | **21** | **17** | **12** | **4** | **22** | **19** | **8** | **4** | **9** | **5** | **6** | **3** |
| % of the total species |  | 84 | 68 | 48 | 16 | 88 | 76 | 32 | 16 | 36 | 20 | 24 | 12 |
| No. of endemic species* |  | 1 | 1 | 0 | 0 | 2 | 2 | 0 | 0 | 1 | 1 | 0 | 0 |

*Species not found in the other salt marsh zones and experimental systems studied.

**Table S3.** Pairwise differences between the Collembola, Oribatida and Mesostigmata communities in the experimental Spiekeroog Island (SI) and artificial island (AI) systems and those in the control system on the Spiekeroog Island (Ctr) in upper salt marsh (USM), lower salt marsh (LSM) and pioneer zone (PZ).

| **Microarthropod taxa** | **Pairs of communities** | **Statistical parameters** | | | Mahalanobis distance | |
| --- | --- | --- | --- | --- | --- | --- |
|  |  | F.model | R^2^ | p-value (“BH”adjusted) |  |  |
| Collembola | Ctr - USM \| SI - USM | **3.71** | **0.27** | **0.009** | | 0.67 |
|  | Ctr - USM \| AI - USM | **2.52** | **0.26** | **0.02** | | 1.40 |
|  | AI - USM \| SI - USM | 1.72 | 0.19 | 0.10 | | 1.88 |
|  | Ctr - LSM \| SI - LSM | **5.00** | **0.36** | **0.009** | | 1.98 |
|  | Ctr - LSM \| AI - LSM | **2.24** | **0.20** | **0.03** | | 1.53 |
|  | AI - LSM \| SI - LSM | 1.89 | 0.19 | 0.15 | | 2.55 |
|  | Ctr - PZ \| SI - PZ | 0.41 | 0.04 | 0.69 | | 0.74 |
|  | Ctr - PZ \| AI - PZ | **4.56** | **0.36** | **0.013** | | 0.94 |
|  | AI - PZ \| SI - PZ | **4.13** | **0.34** | **0.02** | | 1.31 |
| Oribatida | Ctr - USM \| SI - USM | 2.34 | 0.19 | 0.07 | | 0.29 |
|  | Ctr - USM \| AI - USM | - | - | - | | - |
|  | SI - USM \| AI - USM | - | - | - | | - |
|  | Ctr - LSM \| SI - LSM | **4.12** | **0.29** | **0.05** | | 1.08 |
|  | Ctr - LSM \| AI - LSM | 2.97 | 0.33 | 0.11 | | 1.26 |
|  | AI - LSM \| SI - LSM | 6.88 | 0.53 | 0.07 | | 0.28 |
|  | Ctr - PZ \| SI - PZ | 0.44 | 0.08 | 0.91 | | 0.16 |
|  | Ctr - PZ \| AI - PZ | 4.17 | 0.58 | 0.24 | | 2.94 |
|  | AI - PZ \| SI - PZ | 5.59 | 0.74 | 0.29 | | 3.03 |
| Mesostigmata | Ctr - USM \| SI - USM | 1.59 | 0.14 | 0.21 | | 0.17 |
|  | Ctr - USM \| AI - USM | **4.83** | **0.41** | **0.03** | | 3.01 |
|  | AI - USM \| SI - USM | **3.11** | **0.31** | **0.03** | | 3.08 |
|  | Ctr - LSM \| SI - LSM | 2.02 | 0.17 | 0.10 | | 0.75 |
|  | Ctr - LSM \| AI - LSM | **4.45** | **0.33** | **0.02** | | 1.77 |
|  | AI - LSM \| SI - LSM | **2.67** | **0.23** | **0.04** | | 1.49 |
|  | Ctr - PZ \| SI - PZ | 0.47 | 0.05 | 0.79 | | 0.36 |
|  | Ctr - PZ \| AI - PZ | **2.89** | **0.32** | **0.05** | | 0.68 |
|  | AI - PZ \| SI - PZ | 2.03 | 0.29 | 0.07 | | 1.03 |

**Table S4.** Colonization success scores of the Collembola, Oribatida, and Mesostigmata communities in the Spiekeroog Island (SI) and artificial island (AI) systems, calculated as the ratio of the presence-absence (PA) scores of individual taxa in each plot of the experimental salt marsh systems to the respective scores in the control plots of upper salt marsh (USM), lower salt marsh (LSM) and pioneer zone (PZ).

| Exp. systems | Salt  marsh Zones | Plot no. | **Collembola** | | | **Oribatida** | | | **Mesostigmata** | | |
| --- | --- | --- | --- | --- | --- | --- | --- | --- | --- | --- | --- |
|  |  |  | PA score of each plot | PA scores of respective Control plots | Colonization success scores | PA scores of each plot | PA scores of respective Control plots | Colonization success scores | PA scores of each plot | PA scores of respective Control plots | Colonization success scores |
| **SI** | USM | SI_USM_P1 | 4 | 4 | 1 | 3 | 4 | 0.75 | 4 | 3 | 1.33 |
|  |  | SI_USM_P2 | 3 | 3 | 1 | 3 | 4 | 0.75 | 4 | 4 | 1 |
|  |  | SI_USM_P3 | 3 | 4 | 0.75 | 2 | 2 | 1 | 4 | 3 | 1.33 |
|  |  | SI_USM_P4 | 4 | 4 | 1 | 3 | 3 | 1 | 4 | 4 | 1 |
|  |  | SI_USM_P5 | 4 | 4 | 1 | 4 | 4 | 1 | 3 | 4 | 0.75 |
|  |  | SI_USM_P6 | 4 | 4 | 1 | 4 | 4 | 1 | 4 | 4 | 1 |
|  | LSM | SI_LSM_P1 | 4 | 4 | 1 | 4 | 4 | 1 | 4 | 4 | 1 |
|  |  | SI_LSM_P2 | 4 | 4 | 1 | 3 | 4 | 0.75 | 3 | 4 | 0.75 |
|  |  | SI_LSM_P3 | 1 | 4 | 0.25 | 4 | 4 | 1 | 4 | 4 | 1 |
|  |  | SI_LSM_P4 | 3 | 4 | 0.75 | 2 | 2 | 1 | 4 | 4 | 1 |
|  |  | SI_LSM_P5 | 0 | 4 | 0 | 2 | 2 | 1 | 3 | 4 | 0.75 |
|  |  | SI_LSM_P6 | 3 | 4 | 0.75 | 1 | 2 | 0.5 | 4 | 4 | 1 |
|  | PZ | SI_PZ_P1 | 2 | 4 | 0.5 | 1 | 2 | 0.5 | 4 | 4 | 1 |
|  |  | SI_PZ_P2 | 4 | 4 | 1 | 1 | 3 | 0.33 | 4 | 4 | 1 |
|  |  | SI_PZ_P3 | 4 | 4 | 1 | 2 | 3 | 0.67 | 4 | 3 | 1.33 |
|  |  | SI_PZ_P4 | 4 | 4 | 1 | 0 | 1 | 0 | 1 | 4 | 0.25 |
|  |  | SI_PZ_P5 | 3 | 4 | 0.75 | - | - | - | 0 | 1 | 0 |
|  |  | SI_PZ_P6 | 4 | 4 | 1 | - | - | - | 3 | 3 | 1 |
| **AI** | USM | AI_USM_P1 | 1 | 3 | 0.33 | 0 | 2 | 0 | 3 | 3 | 1 |
|  |  | AI_USM_P2 | 1 | 4 | 0.25 | 0 | 3 | 0 | 2 | 4 | 0.5 |
|  |  | AI_USM_P3 | 3 | 4 | 0.75 | 0 | 4 | 0 | 2 | 4 | 0.5 |
|  |  | AI_USM_P4 | 0 | 4 | 0 | 0 | 4 | 0 | 0 | 3 | 0 |
|  |  | AI_USM_P5 | 0 | 4 | 0 | 0 | 4 | 0 | 0 | 4 | 0 |
|  |  | AI_USM_P6 | 0 | 4 | 0 | 0 | 4 | 0 | 0 | 4 | 0 |
|  | LSM | AI_LSM_P1 | 3 | 4 | 0.75 | 0 | 4 | 0 | 4 | 4 | 1 |
|  |  | AI_LSM_P2 | 1 | 4 | 0.25 | 0 | 4 | 0 | 4 | 4 | 1 |
|  |  | AI_LSM_P3 | 2 | 4 | 0.5 | 0 | 2 | 0 | 4 | 4 | 1 |
|  |  | AI_LSM_P4 | 3 | 4 | 0.75 | 0 | 2 | 0 | 3 | 4 | 0.75 |
|  |  | AI_LSM_P5 | 4 | 4 | 1 | 1 | 4 | 0.25 | 4 | 4 | 1 |
|  |  | AI_LSM_P6 | 0 | 4 | 0 | 1 | 2 | 0.5 | 0 | 4 | 0 |
|  | PZ | AI_PZ_P1 | 1 | 4 | 0.25 | 1 | 2 | 0.5 | 1 | 4 | 0.25 |
|  |  | AI_PZ_P2 | 2 | 4 | 0.5 | 0 | 3 | 0 | 0 | 3 | 0 |
|  |  | AI_PZ_P3 | 1 | 4 | 0.25 | 0 | 1 | 0 | 4 | 1 | 4***** |
|  |  | AI_PZ_P4 | 1 | 4 | 0.25 | 0 | 3 | 0 | 0 | 4 | 0 |
|  |  | AI_PZ_P5 | 0 | 4 | 0 | - | - | - | 0 | 4 | 0 |
|  |  | AI_PZ_P6 | 0 | 4 | 0 | - | - | - | 0 | 3 | 0 |

***** Outlier (removed from the analysis)

**Figure S1**. Colonization of upper salt marsh (USM), lower salt marsh (LSM) and pioneer zone (PZ) of the natural salt marsh control system (Ctr) and the experimental Spiekeroog Island (SI) and artificial island (AI) systems by Collembola, Oribatida and Mesostigmata species. The size of the bubbles indicates the relative abundance of the species in the treatment.

**
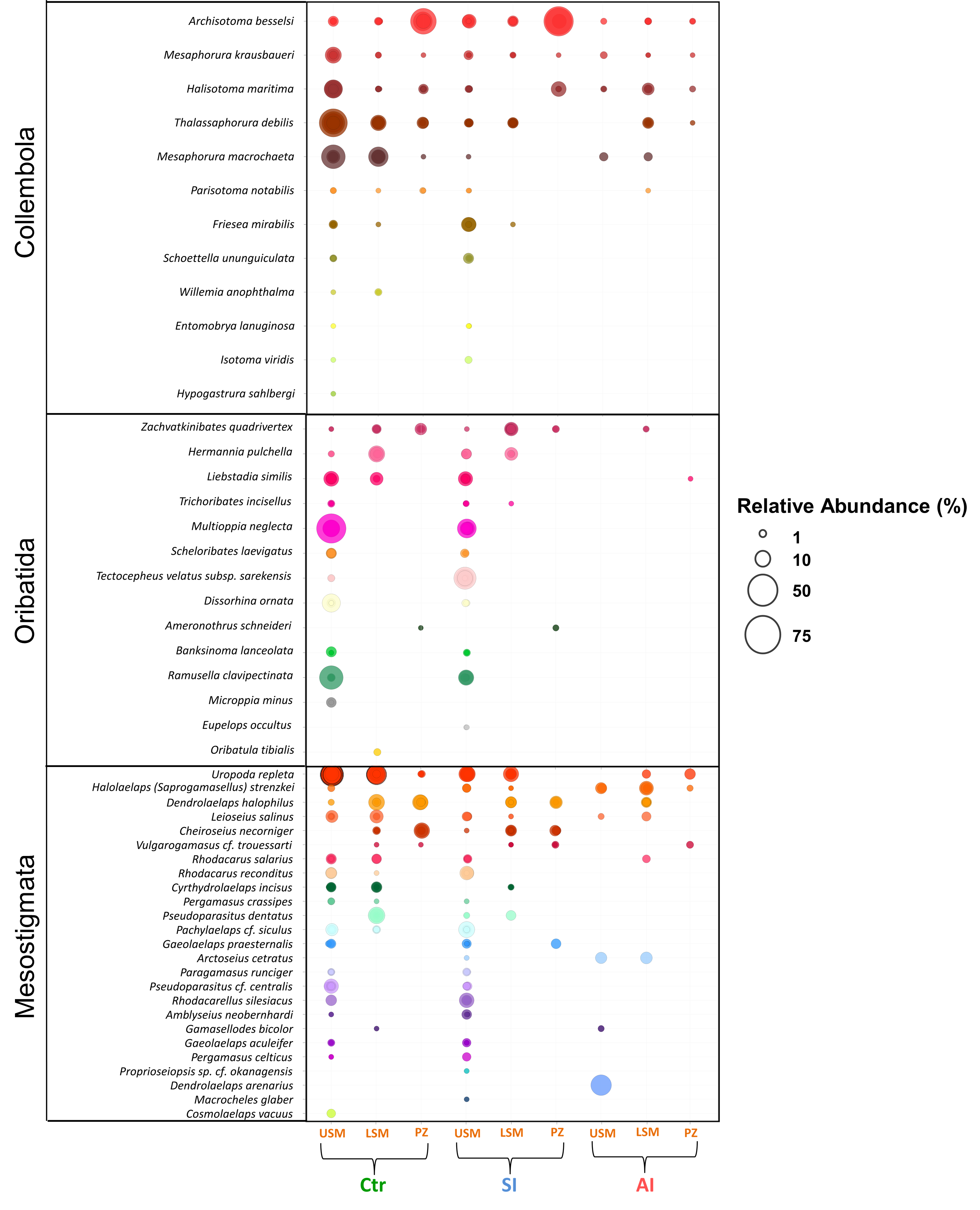
**

**Figure S2**. Relationships between colonization success rates and community-weighted mean (CWM) body size of microarthropods (Collembola, Oribatida and Mesostigmata) as indicated by the Kendall's **τ** correlation coefficient and p value.


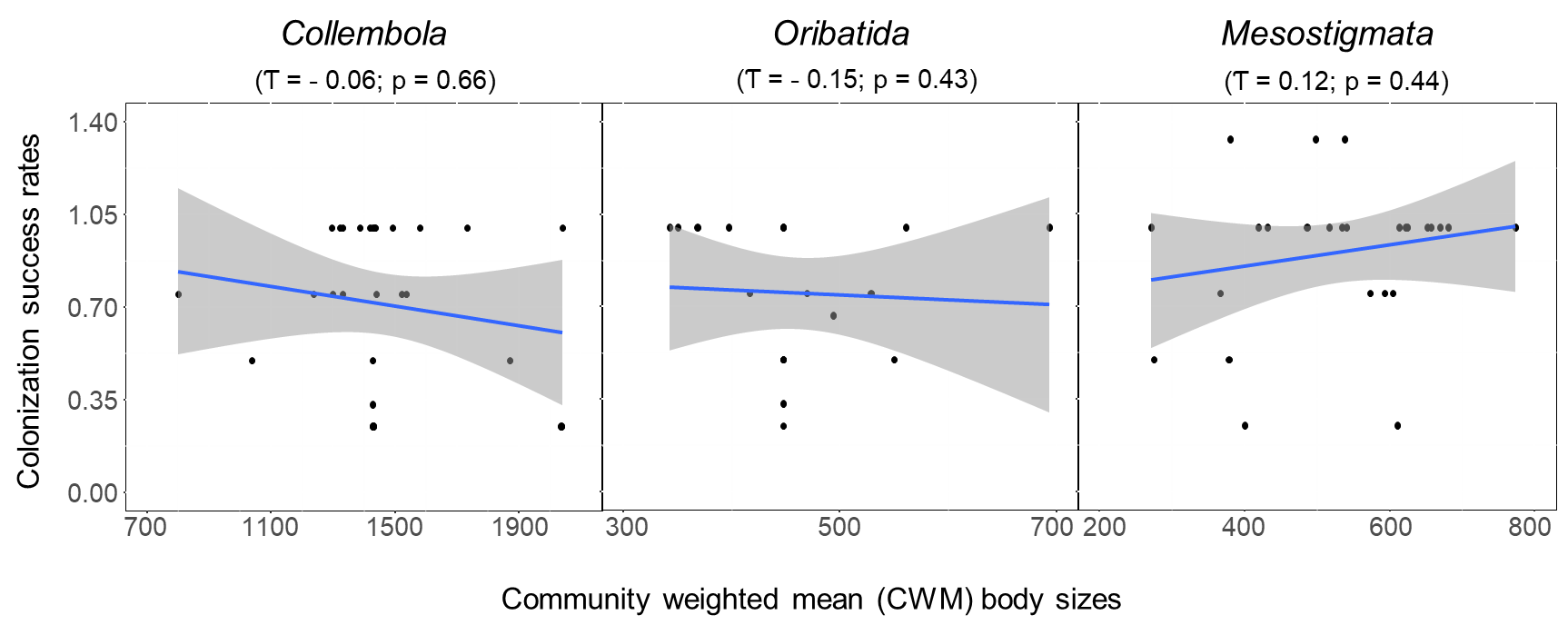


**References**

Karg, W. (1989). Acari (Acarina), Milben. Parasitiformes (Anactinochaeta). Uropodina Kramer, Schildkrötenmilben, F. Dahl (Ed.), Die Tierwelt Deutschlands, Gustav Fischer, Jena.

Karg, W. (1993). Acari (Acarina), Milben. Parasitiformes (Anactinochaeta). Cohors Gamasina Leach. Raubmilben, F. Dahl (Ed.), Die Tierwelt Deutschlands (second ed.), Gustav Fischer, Jena.

Weigmann, G. (2006). Hornmilben (Oribatida) (Die Tierwelt Deutschlands). Goecke & Evers, ISBN: 3-937783-18-0
